# Supplementary figures and images for: Set2 histone methyltransferase regulates transcription coupled-nucleotide excision repair in yeast
Source: PLoS Genet. 2022 Mar 9;18(3):e1010085. doi: 10.1371/journal.pgen.1010085 (PMC8936446; doi:10.1371/journal.pgen.1010085)

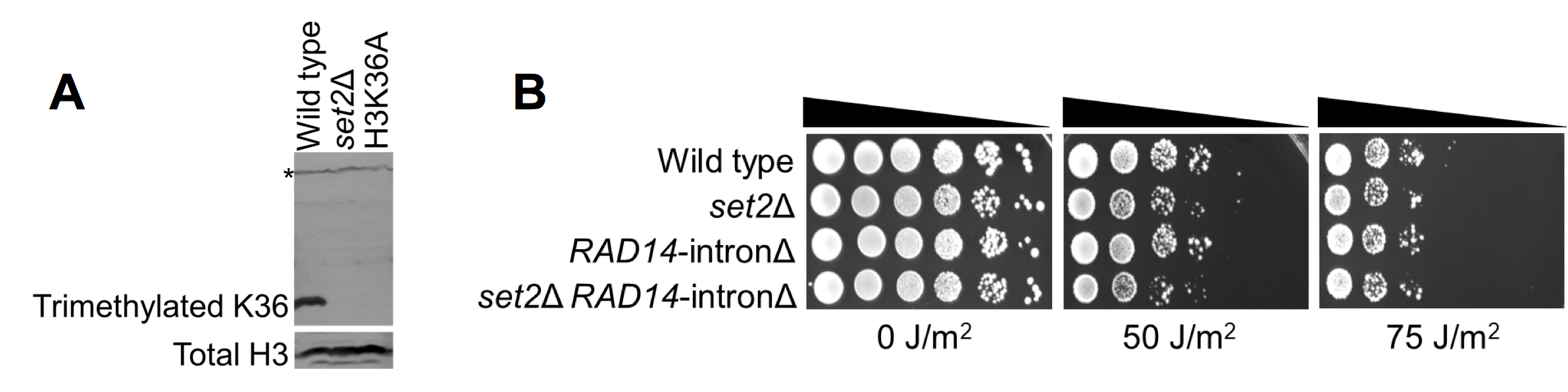

Supplement: S1 Fig — (A) Western blot confirming that deletion of SET2 or mutation of H3K36 eliminates H3K36 methylation. Western blot analysis using anti-H3K36me3 or anti-H3 (loading control) was used to probe total protein extracts isolated from the indicated yeast strains. Asterisk (*) indicates a nonspecific cross-reacting band recognized by the anti-H3K36me3 antibody. (B) UV sensitivity assay indicating that the intron in the RAD14 gene is not required for set2Δ UV sensitivity. UV sensitivity assays were performed using the indicated UVC doses for the specified yeast strains. (TIF) [file pgen.1010085.s001.tif]

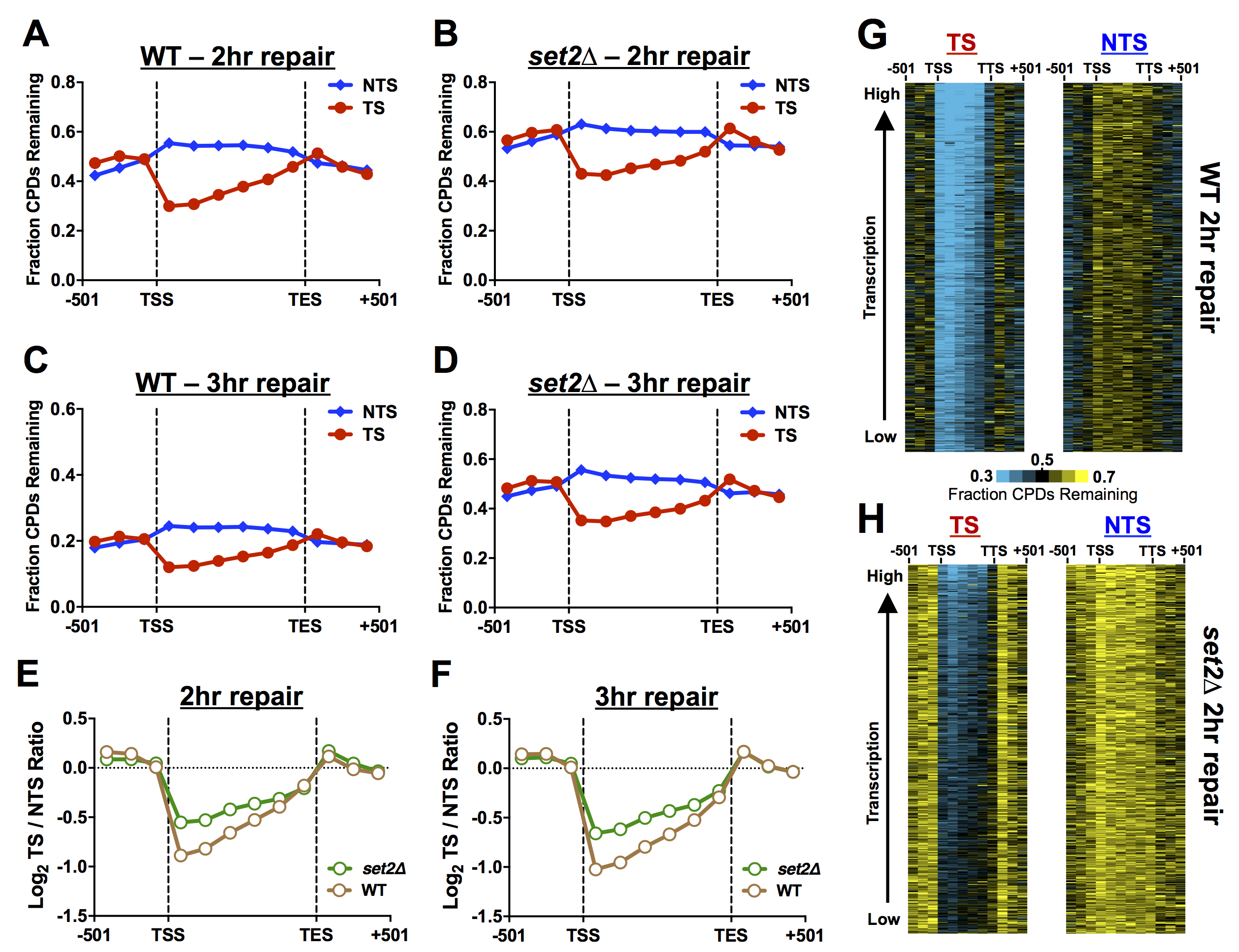

Supplement: S2 Fig — (A-D) Bin plot analysis of the average fraction of CPDs remaining after 2 or 3 hours of repair was plotted from the CPD-seq data along the transcribed strand (TS) and non-transcribed strand (NTS) of ~5000 yeast genes in WT (A,C) and set2Δ (B,D) mutant cells. Each gene was divided into six equally sized bins and the fraction of unrepaired CPDs at the indicated time point relative to 0hr time point was plotted. Fraction of CPDs remaining in three bins (167 bp in length) upstream of transcription start site (TSS), as well as downstream of transcription end site (TES) was also plotted. (E-F) Log2 ratio of unrepaired CPDs on the TS relative to the NTS in the WT and set2Δ cells at 2hr (E) or 3hr (F) repair time points. (G-H) Gene plot analysis of WT and set2Δ cells depicting the fraction of CPDs remaining following 2hr of repair (relative to 0hr) for both the TS and NTS. Genes are ordered based on their published transcription frequency [42]. (TIF) [file pgen.1010085.s002.tif]

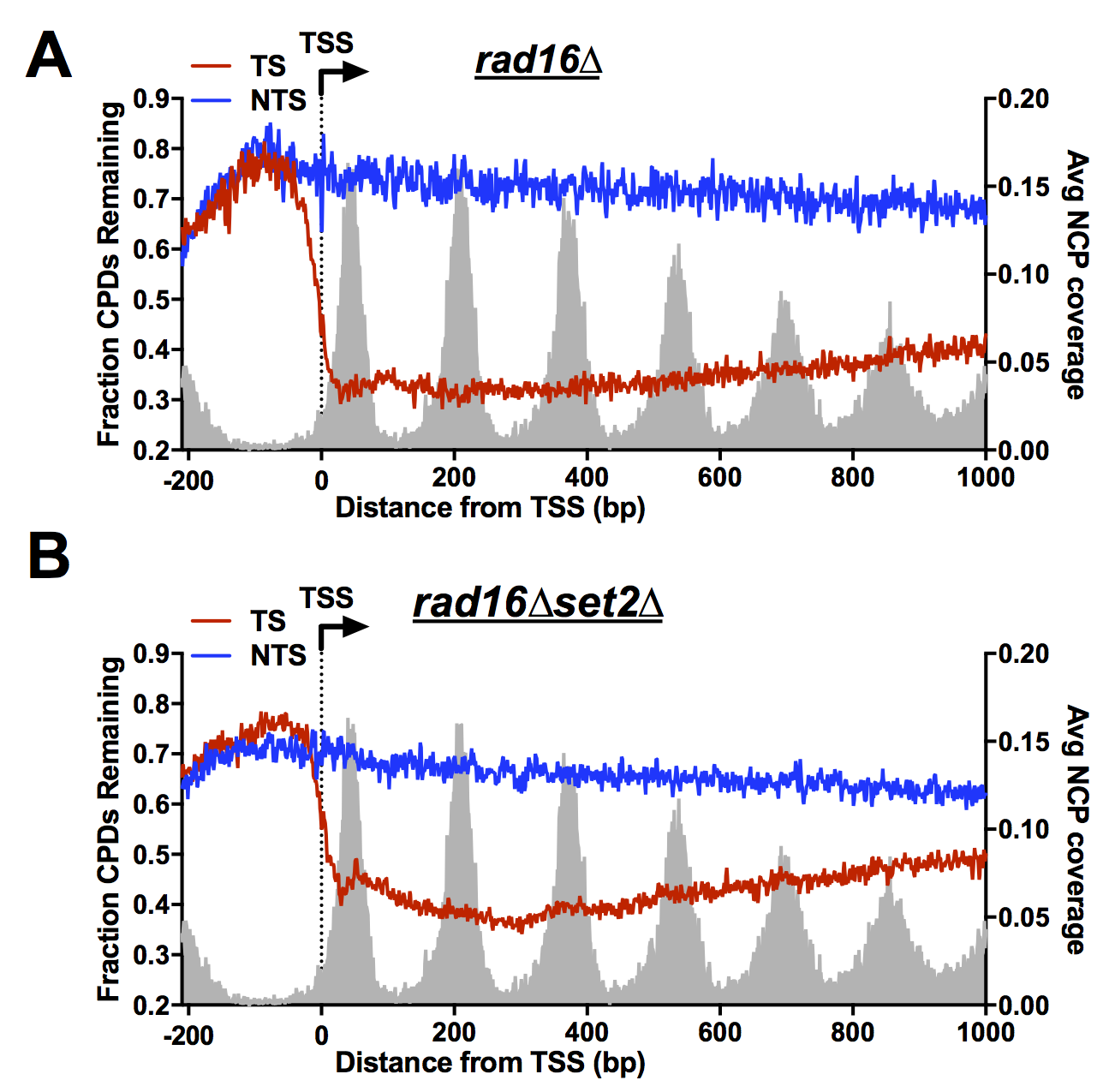

Supplement: S3 Fig — (A-B) High-resolution analysis of the CPD-seq data showing the fraction of CPDs remaining after 2hr repair in rad16Δ (A) and rad16Δset2Δ (B) mutant cells around the TSS of ~5200 yeast genes. Average nucleosome coverage from [77] is shown for reference. (TIF) [file pgen.1010085.s003.tif]

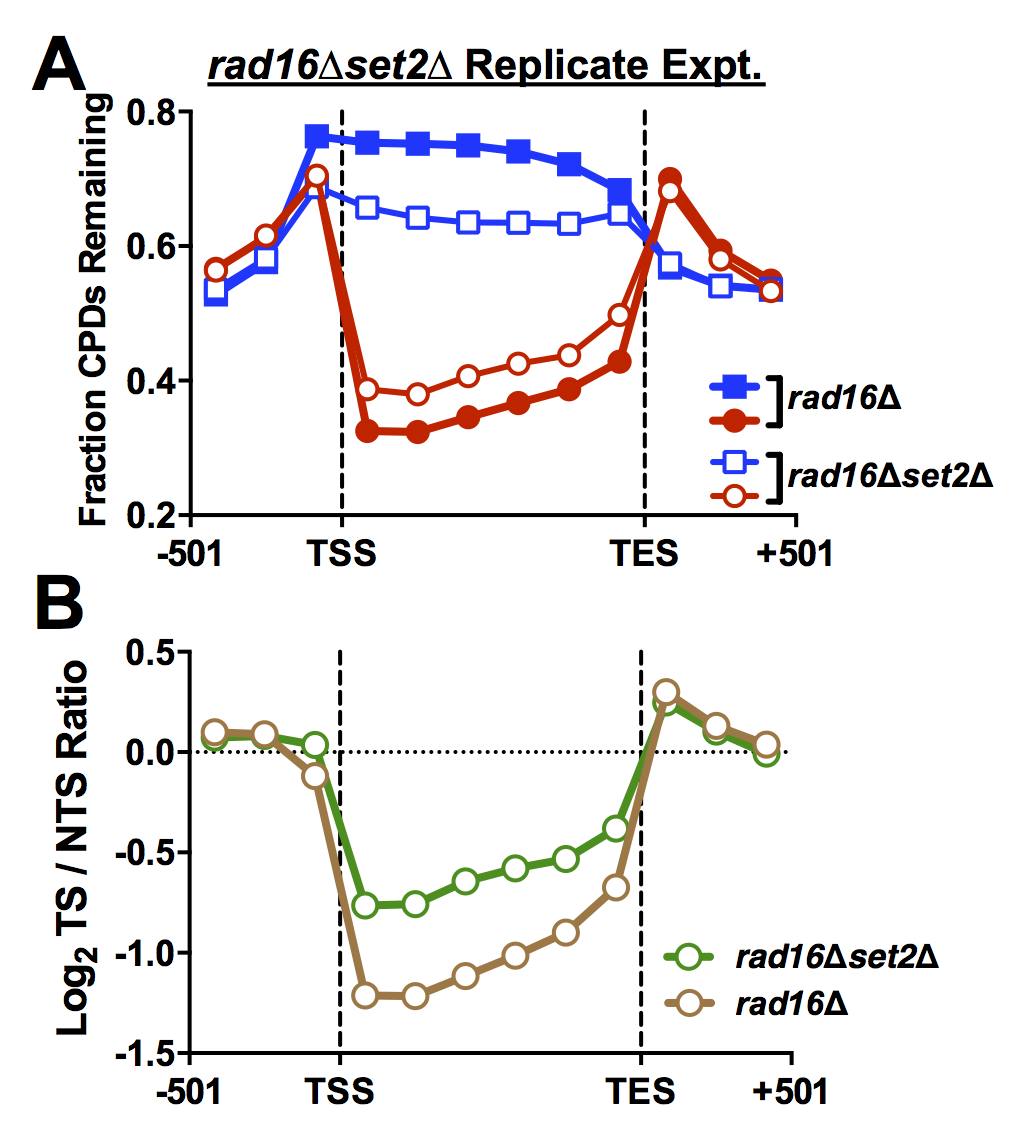

Supplement: S4 Fig — (A) Bin plot analysis of CPD-seq data for rad16Δ (data from [39]) and replicate rad16Δset2Δ experiment. Each gene was divided into six equally sized bins and the fraction of unrepaired CPDs at the indicated time point relative to 0hr time point was plotted. Fraction of CPDs remaining in three bins (167 bp in length) upstream of transcription start site (TSS), as well as downstream of transcription end site (TES) was also plotted. CPD-seq data for rad16Δset2Δ mutant was scaled so that the median ratio of fraction of CPDs remaining for the rad16Δset2Δ relative to the rad16Δ control for the upstream and downstream intergenic DNA bins was set to 1. Transcribed strand (TS) is in red; non-transcribed strand (NTS) is in blue. (B) Quantification of repair asymmetry in replicate rad16Δset2Δ relative to the rad16Δ control, calculated as the log2 ratio of unrepaired CPDs on the TS relative to the NTS for each bin. (TIF) [file pgen.1010085.s004.tif]

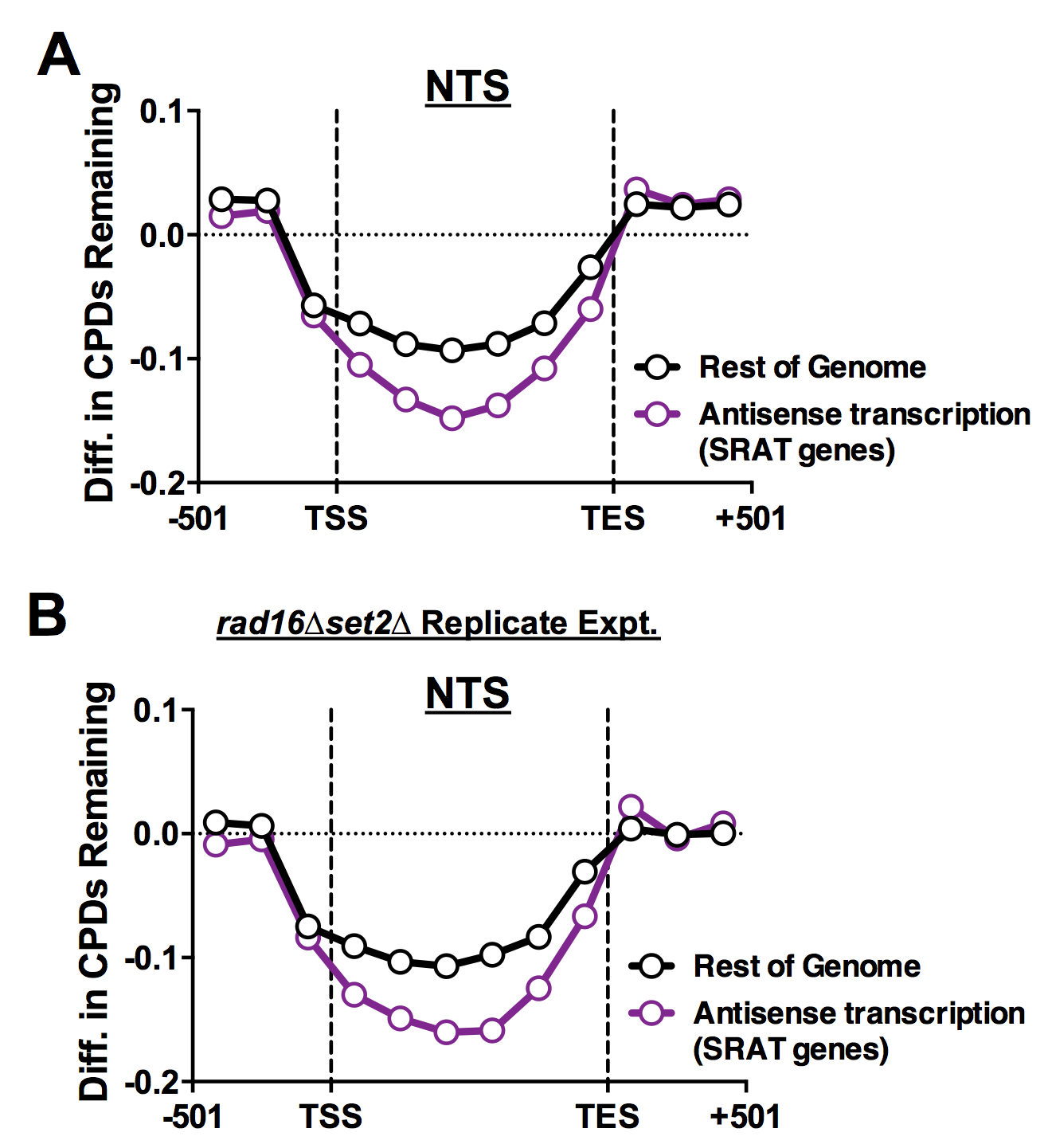

Supplement: S5 Fig — (A) Quantification of difference in CPDs remaining (after 2hr repair) along the NTS between rad16Δset2Δ double mutant and rad16Δ single mutant for genes associated with polyA SRAT (i.e., antisense transcripts) and those that are not (rest of genome). (B) Same as panel A, except for rad16Δset2Δ replicate experiment. Replicate CPD-seq data for rad16Δset2Δ mutant was scaled so that the overall median ratio of fraction of CPDs remaining for the upstream and downstream intergenic DNA bins was set to 1. (TIF) [file pgen.1010085.s005.tif]

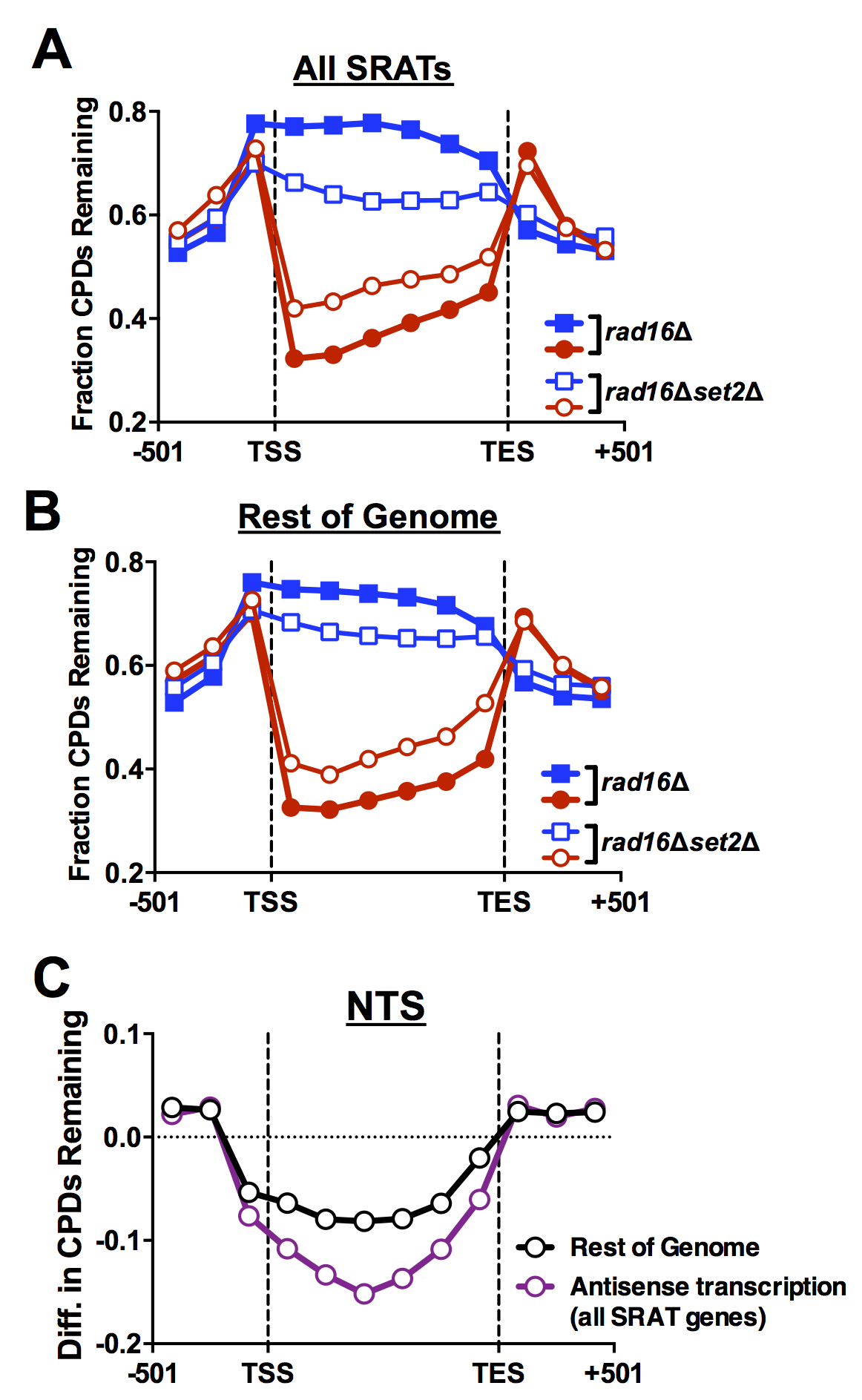

Supplement: S6 Fig — (A) Bin plot analysis of CPD-seq data for rad16Δset2Δ double mutant and rad16Δ control (from [39]) for all genes associated with a Set2-repressed antisense transcript (all SRATs; not just polyA SRATs). Each gene was divided into six equally sized bins and the fraction of unrepaired CPDs following 2hr repair relative to 0hr time point was plotted. Fraction of CPDs remaining in three bins (167 bp in length) upstream of transcription start site (TSS), as well as downstream of transcription end site (TES) was also plotted. Transcribed strand (TS) is in red; non-transcribed strand (NTS) is in blue. SRAT gene list from [46]. (B) Same as panel A, except for genes not associated with an SRAT. (C) Quantification of difference in CPDs remaining between rad16Δset2Δ double mutant relative to the rad16Δ control along the NTS for each bin. Genes associated with an SRAT are depicted in purple; non-SRAT genes are shown in black. (TIF) [file pgen.1010085.s006.tif]

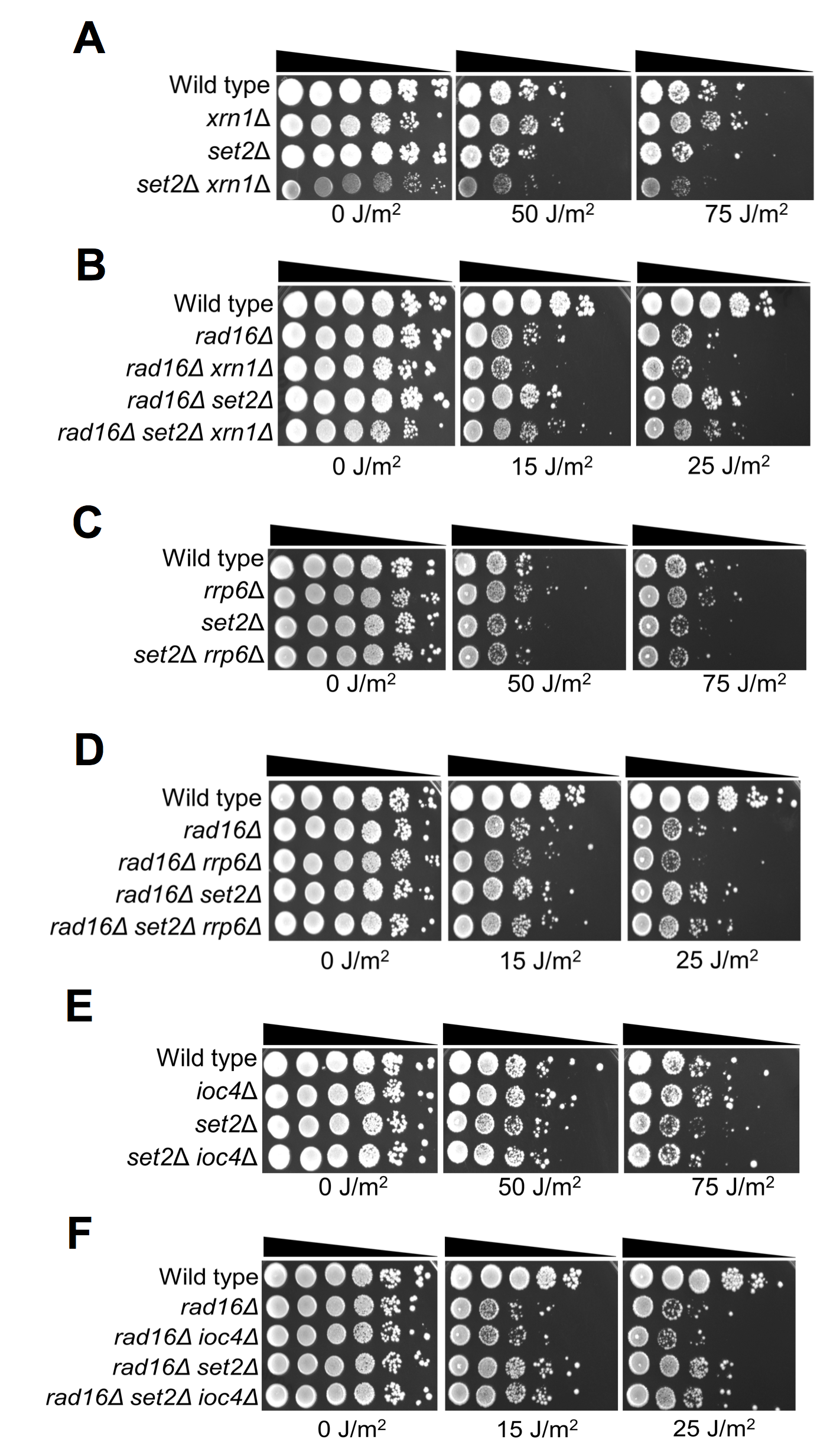

Supplement: S7 Fig — (A-D) UV sensitivity assays indicate that deleting XRN1 or RRP6 exonucleases to stabilize the SRAT transcripts does not modulate set2Δ UV sensitivity in WT background (A,C) or UV resistance in a rad16Δ mutant background (B,D). UV sensitivity assays were performed using the indicated UVC doses for the specified yeast strains. (E,F) UV sensitivity assays indicates that Ioc4 (subunit of the Isw1b complex) does not mimic set2Δ UV sensitivity (E) or modulate UV resistance in rad16Δ mutant background (F). (TIF) [file pgen.1010085.s007.tif]

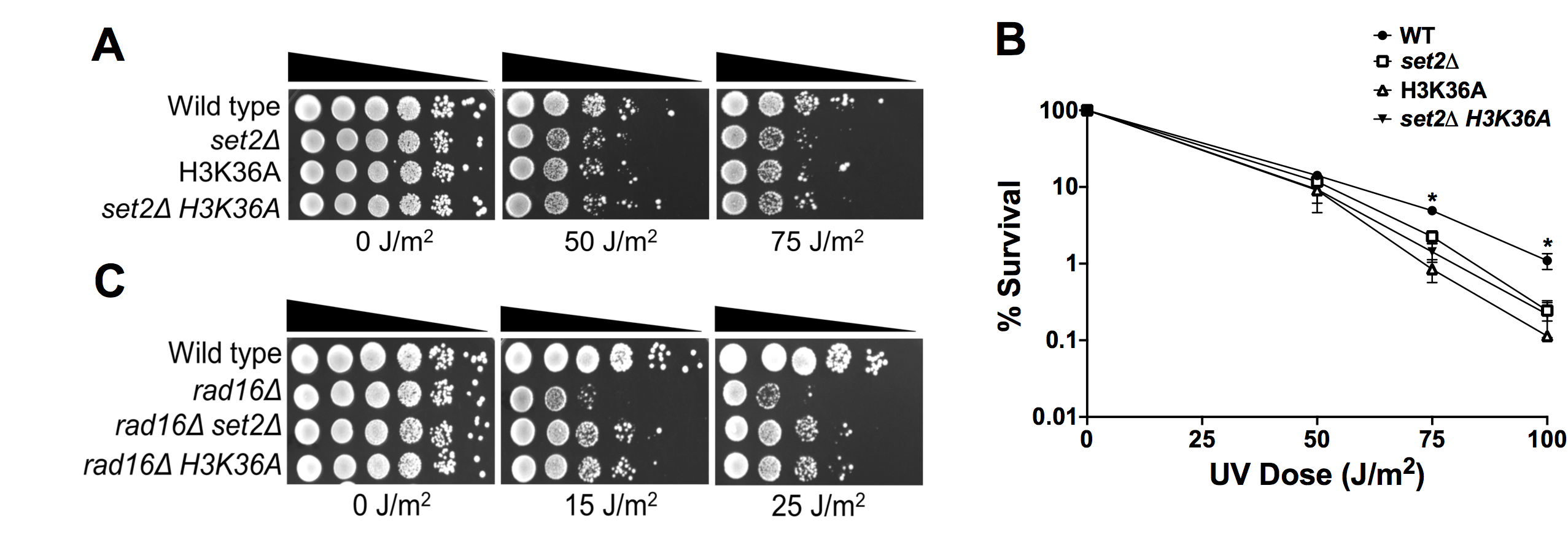

Supplement: S8 Fig — (A) UV sensitivity assay showing sensitivity of set2Δ mutant (deleted in histone WT background) is similar to that of H3K36A mutant. UV sensitivity assays were performed using the specified UVC doses for the indicated yeast strains. (B) Graph of data for a quantitative UV sensitivity assay for the indicated yeast strains. Mean ± SEM is plotted for n = 3 replicates. Asterisks (*) indicate significant difference in UV sensitivity between WT and mutant strains (P < 0.05), using two-sided t-test. Source data is in S3 Data. (C) Same as panel A, except showing that UV resistance of set2Δ mutant (deleted in histone WT rad16Δ background) is similar to that of H3K36A mutant in a rad16Δ background. (TIF) [file pgen.1010085.s008.tif]

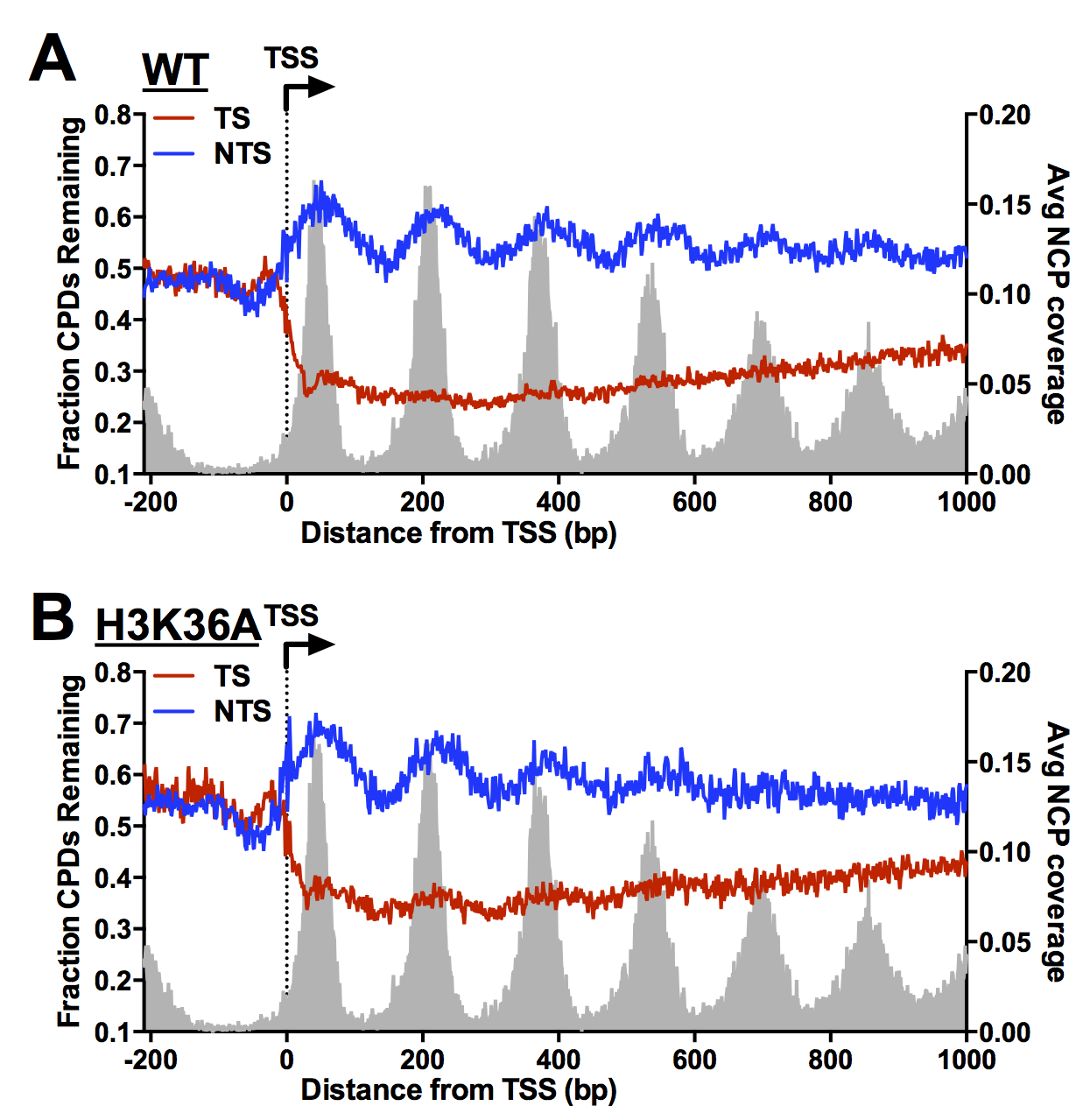

Supplement: S9 Fig — (A-B) High-resolution analysis of the CPD-seq data showing the fraction of CPDs remaining after 2hr repair relative to the 0hr time point in histone WT (A) and H3K36A (B) mutant cells around the transcription start site (TSS) of ~5200 yeast genes. CPD-seq data was normalized using overall repair at 2hr from alkaline gel analysis of histone WT and H3K36A mutant. Average nucleosome coverage from [77] is shown for reference. (TIF) [file pgen.1010085.s009.tif]

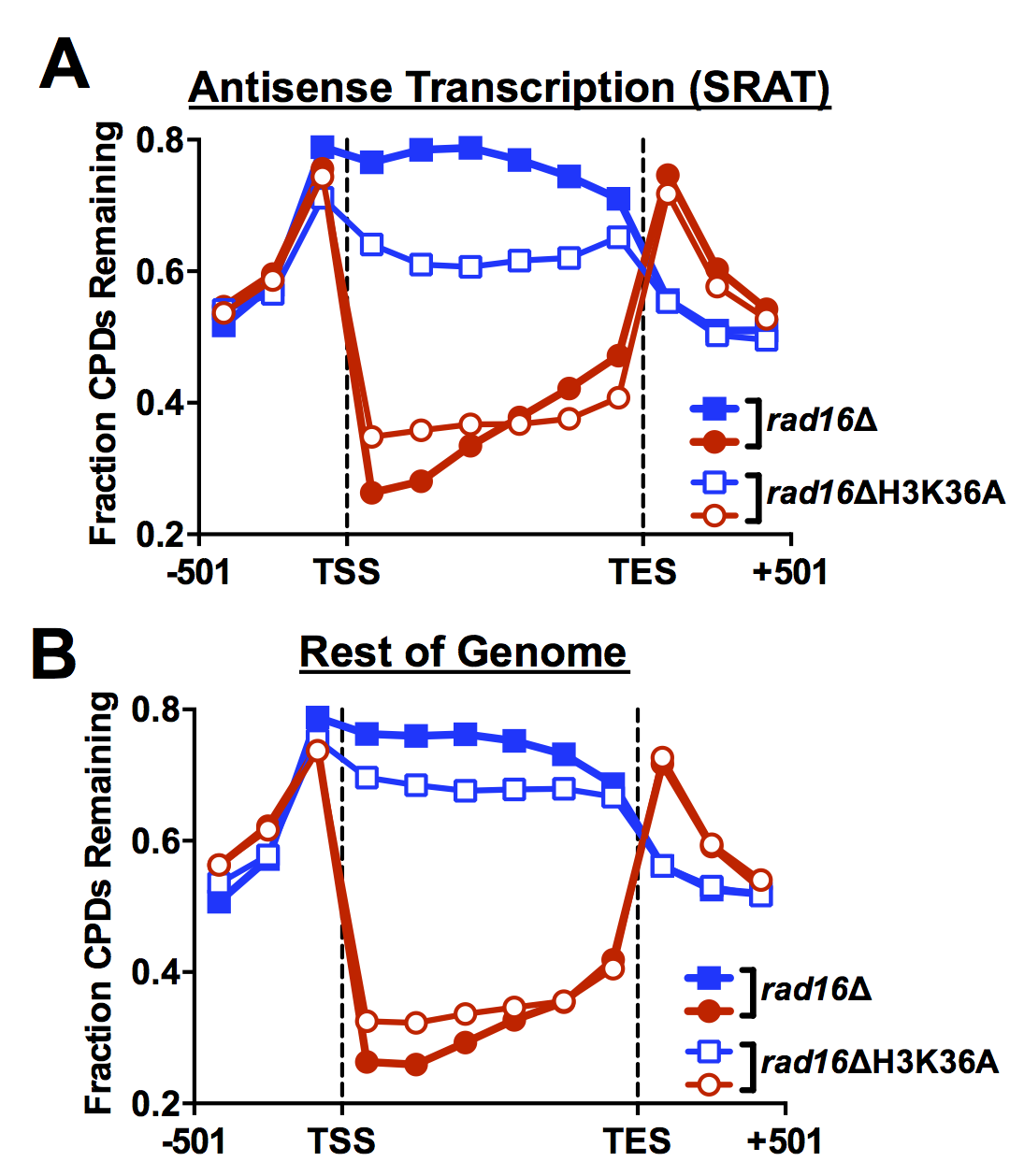

Supplement: S10 Fig — (A) Bin plot analysis of CPD-seq data for rad16ΔH3K36A double mutant and rad16Δ control (histone WT) for all genes associated with a polyA Set2-repressed antisense transcript (SRAT). Each gene was divided into six equally sized bins and the fraction of unrepaired CPDs following 2hr repair relative to 0hr time point was plotted. Fraction of CPDs remaining in three bins (167 bp in length) upstream of transcription start site (TSS), as well as downstream of transcription end site (TES) was also plotted. Transcribed strand (TS) is in red; non-transcribed strand (NTS) is in blue. SRAT gene list from [46]. (B) Same as panel A, except for genes not associated with a polyA SRAT. CPD-seq data for rad16ΔH3K36A mutant was scaled so that the overall median ratio of fraction of CPDs remaining for the rad16ΔH3K36A mutant relative to the rad16Δ (histone WT) control for the upstream and downstream intergenic DNA bins was set to 1. (TIF) [file pgen.1010085.s010.tif]

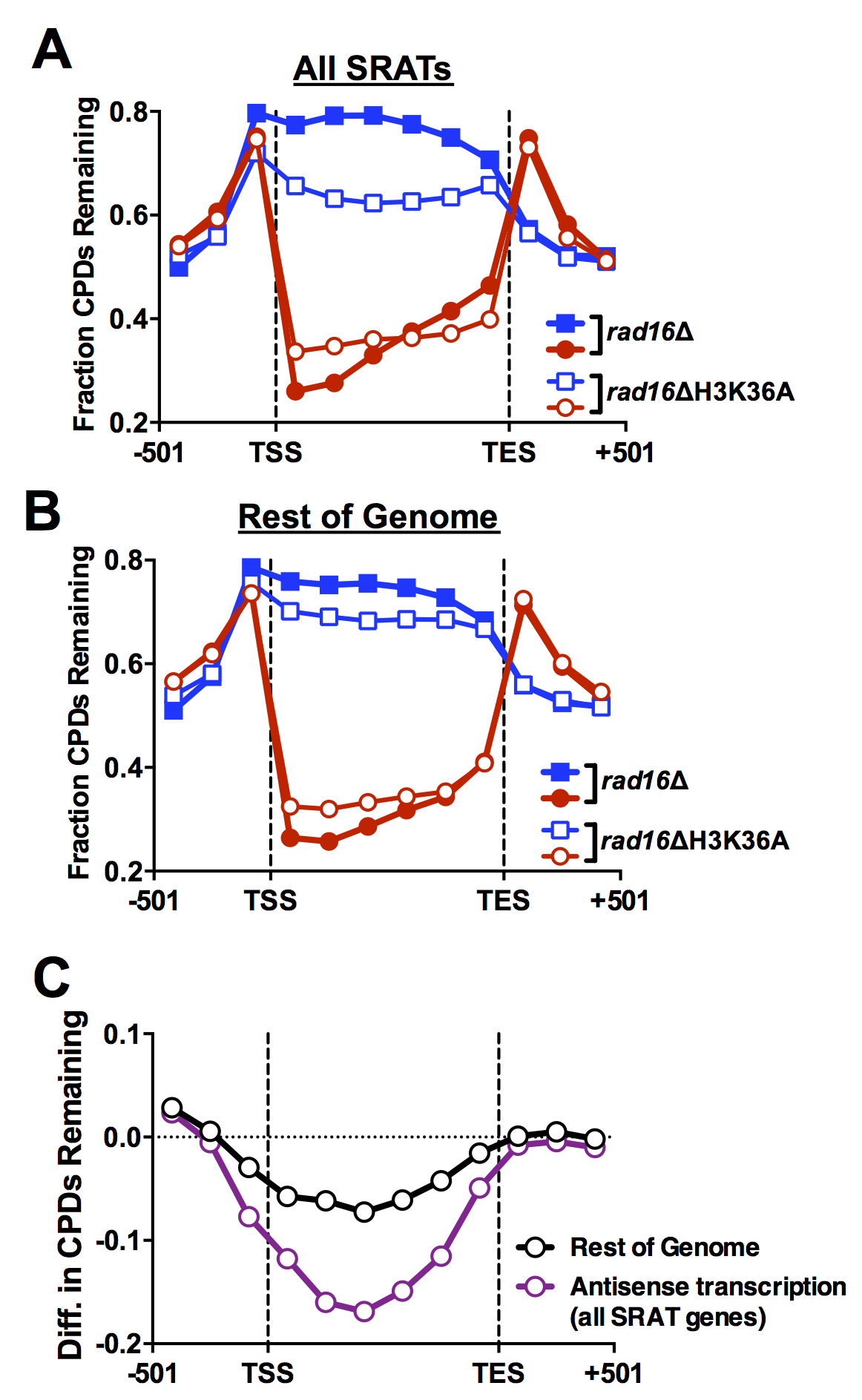

Supplement: S11 Fig — (A) Bin plot analysis of CPD-seq data for rad16ΔH3K36A double mutant and rad16Δ (histone WT) control for all genes associated with a Set2-repressed antisense transcript (SRAT; not just polyA SRATs). Each gene was divided into six equally sized bins and the fraction of unrepaired CPDs following 2hr repair relative to 0hr time point was plotted. Fraction of CPDs remaining in three bins (167 bp in length) upstream of transcription start site (TSS), as well as downstream of transcription end site (TES) was also plotted. Transcribed strand (TS) is in red; non-transcribed strand (NTS) is in blue. SRAT gene list from [46]. (B) Same as panel A, except for genes not associated with an SRAT. (C) Quantification of difference in CPDs remaining between rad16ΔH3K36A double mutant relative to the rad16Δ (histone WT) control along the NTS for each bin. Genes associated with an SRAT are depicted in purple; non-SRAT genes are shown in black. CPD-seq data for rad16ΔH3K36A mutant was scaled so that the overall median ratio of fraction of CPDs remaining for the rad16ΔH3K36A mutant relative to the rad16Δ (histone WT) control for the upstream and downstream intergenic DNA bins was set to 1. (TIF) [file pgen.1010085.s011.tif]
