# Supplementary material for: Set2 histone methyltransferase regulates transcription coupled-nucleotide excision repair in yeast
Source: PLoS Genet. 2022 Mar 9;18(3):e1010085. doi: 10.1371/journal.pgen.1010085 (PMC8936446; doi:10.1371/journal.pgen.1010085)
Supplement: Attachment — Submitted filename: Selvam et al review.pdf [file pgen.1010085.s015.pdf]

In this manuscript, Selvam *et al.* investigate the role of the histone H3K36 methyltransferase Set2 in nucleotide excision repair (NER) in the budding yeast *S. cerevisiae*. Previous work demonstrated that H3K36 methylation (H3K36me) suppresses intragenic transcription by recruiting the Rpd3-containing histone deacetylase complex. This investigation further explores the importance of Set2 and H3K36me to UV-induced DNA damage repair by specifically measuring transcription coupled- and global genomic-NER (TC-NER and GG-NER, respectively). Their results suggest that H3K36me plays opposing roles in the NER pathway by stimulating TC-NER while antagonizing GG-NER. The authors hypothesize that observed increases in non-transcribed strand repair observed in the *set2Δ* mutants are due to an increase in cryptic antisense transcription of this strand. They test this hypothesis by genetically dissecting the cryptic transcription pathway through deletion of either Eaf3 or Dot1 and find that eliminating factors involved in cryptic antisense transcription protects cells against Rad16-dependent UV-induced cell death. Together, their data are interesting and provide evidence that Set2 may act as a molecular toggle that can regulate the rate of TC- and GG-NER following UV damage. In addition to the interesting data, the manuscript has many strengths including the implementation of a CPD-seq methodology that was developed in their laboratory.

Despite these strengths, the authors' model of Set2-mediated H3K36me as the positive regulatory factor for TC-NER (and antagonistic function for GG-NER) should be directly examined. The authors should provide additional data directly testing this model by either 1) measuring the histone H3K36me profile of cells following UV-damage (using Western blot analyses, ChIP-seq, or another ChIP-based methodology) and/or 2) testing the role of H3K36me genetically by including a *set2Δ*H3K36A double mutant in their analyses. By directly measuring the role of H3K36me, they may identify non-histone Set2 substrates important for UV-induced DNA damage repair. In addition, these analyses might indicate which level(s) of H3K36me are important for UV damage repair and/or recruitment of the repair machinery. The study would also be significantly strengthened by more rigorous analyses of yeast harboring H3K36A point mutations. CPD-seq for H3K36A, *rad16* H3K36A and *rad26* H3K36A mutant. Other issues with the manuscript can be found below:

1. In Fig. 1, the authors conclude that Set2-mediated H3K36me is required for resistance to UV damage. If this is the case, then one would expect a phenocopy when comparing UV sensitivity of *set2Δ* to H3K36A mutants. As presented, the data are not convincing that the point mutation of H3K36 demonstrates equal UV sensitivity to *set2Δ* mutants. Perhaps this would be more convincing if the data were quantified (as in Fig. 5). This experiment would also be strengthened by inclusion of a *set2Δ*H3K36A double mutant, as discussed above. Furthermore, as the data are presented, the reader cannot make a direct comparison of the *set2Δ* and H3K36A mutant strains since the experiments were performed on separate plates. At a minimum, the authors should repeat the experiment on the same plate to allow direct comparison of *set2Δ* and H3K36A mutants. Finally, the H3K36A mutant was not assayed for the kinetics of DNA repair (panels F and G) and should be included to bolster their conclusions.

2. In Fig. 1F and 1G, the authors quantify alkaline gels to measure global CPD repair conclude that there is a minimal difference in Set2-dependent repair at the 3-h time point. Since TC-NER occurs preferentially over GG-NER, these data seem inconsistent with their model that Set2 is important for TC-NER and antagonizes GG-NER. Some discussion of this result seems warranted.

3. In Fig. 2D and E, the authors hypothesize that differences in Set2-dependent TC-NER should be reflected by plotting CPD-seq data alongside the transcription frequency of yeast genes. The analyses as presented utilizes the transcript levels of wild type yeast for analyses of *set2Δ* mutants. Since Set2 is a known regulator of transcription, the authors should repeat the *set2Δ* CPD repair analyses using transcript levels from *set2Δ* yeast mutants using available data from references 30-34.

4. Fig. 2E shows that the majority of NTS CPDs still remain in the *set2Δ* mutant after 2 hours of repair. This result influences their $\log_2$ ratio analyses of the wild type and *set2Δ* mutant strains. It also suggests that Set2 impacts GG-NER when the wild type repair enzymes are present. Can the authors comment and/or clarify this result in the text? (Is this also related to the discrepancy in Set2 repair as described in Fig. 1G at t=3?) It could support a model in which the delayed TC-NER in Set2 mutants results in a delay to repair the NTS.

5. In Fig. 3 the authors discuss the increase in unrepaired CPDs across the TS in *set2Δ* mutants. However, it seems that Set2 is important for CPD repair in general as there are more unrepaired CPDs across all genomic regions measured. For example, the wild type strain starts at a fraction of CPD remaining=0.5, while *set2Δ* = 0.6. Is the difference they are measuring relative to the starting number or is it more important to consider overall CPD repair? If these curves were presented on the same plot, this conclusion would might be clearer.

MINOR POINTS

1. The title of the manuscript only considers the role of Set2 on TC-NER, yet there are also compelling results regarding the role of Set2 on GG-NER. The authors may wish to consider a title that more accurately depicts their complete dataset.

2. A brief description of CPD-seq in the Results section would assist the reader in interpreting their results.

3. Can the authors clarify why they decided to use CPD-seq at the t=2 h time point? Their previous manuscript describing CPD-seq (Mao et al. 2016) indicates that strand-specific differences in CPD repair can be observed after only 20 minutes. It would be helpful for the reader to include some rationale for their decision on this time point.

4. Are Supplemental Fig. S2G and S2H showing the same data as Fig. 2D and E? It is unclear what the differences are between these datasets (it seems that the Fig S2G and S2H might be from the 3 h. time point).

5. Figs. 4D, 4E, 5A, and 5B line colors are not indicated. I'm assuming that red is TS and blue is NTS as described earlier in the manuscript, but this should be clarified.

6. Fig. 5C needs clarification related to what exactly the authors are measuring. More clearly labeling the figure could provide this clarification.

7. Pg. 10- Citation is missing for the requirement of Rad26 and Rad16 for TC- and GG-NER respectively.

8. Pg. 11- The alkaline gel is indicated as Fig. 1D, but is actually Fig. 1F; Quantification of the data is indicated as Fig. 1E, but is actually Fig. 1G.

9. Pg. 15- The authors state that "there were fewer unrepaired CPDs along the NTS in the *rad16Δset2Δ* double mutant relative to the *rad16* single mutant, indicating that *set2Δ* promotes repair of the NTS in the *rad16* background." This is confusing as it is written, since it is considering the impact of *set2* deletion mutations on Rad16-dependent GG-NER. Perhaps it should be reworded to indicate the impact of Set2 on CPD repair (ie Set2 inhibits repair of the NTS). Similar issues are found throughout the manuscript regarding conclusions about the absence of a gene rather than the function of the gene.
