# Supplementary material for: Set2 histone methyltransferase regulates transcription coupled-nucleotide excision repair in yeast
Source: PLoS Genet. 2022 Mar 9;18(3):e1010085. doi: 10.1371/journal.pgen.1010085 (PMC8936446; doi:10.1371/journal.pgen.1010085)
Supplement: Attachment — Submitted filename: Selvam_etal_ResponsetoReviewers_final.pdf [file pgen.1010085.s017.pdf]

RESPONSE TO REVIEWERS

Reviewer 1:

"In this manuscript, Selvam *et al.* investigate the role of the histone H3K36 methyltransferase Set2 in nucleotide excision repair (NER) in the budding yeast *S. cerevisiae*. Previous work demonstrated that H3K36 methylation (H3K36me) suppresses intragenic transcription by recruiting the Rpd3-containing histone deacetylase complex. This investigation further explores the importance of Set2 and H3K36me to UV-induced DNA damage repair by specifically measuring transcription coupled- and global genomic-NER (TC-NER and GG-NER, respectively). Their results suggest that H3K36me plays opposing roles in the NER pathway by stimulating TCNER while antagonizing GG-NER. The authors hypothesize that observed increases in nontranscribed strand repair observed in the *set2*Δ mutants are due to an increase in cryptic antisense transcription of this strand. They test this hypothesis by genetically dissecting the cryptic transcription pathway through deletion of either Eaf3 or Dot1 and find that eliminating factors involved in cryptic antisense transcription protects cells against Rad16-dependent UV induced cell death. Together, their data are interesting and provide evidence that Set2 may act as a molecular toggle that can regulate the rate of TC- and GG-NER following UV damage. In addition to the interesting data, the manuscript has many strengths including the implementation of a CPD-seq methodology that was developed in their laboratory.

"Despite these strengths, the authors' model of Set2-mediated H3K36me as the positive regulatory factor for TC-NER (and antagonistic function for GG-NER) should be directly examined. The authors should provide additional data directly testing this model by either 1) measuring the histone H3K36me profile of cells following UV-damage (using Western blot analyses, ChIP-seq, or another ChIP-based methodology) and/or 2) testing the role of H3K36me genetically by including a *set2*Δ H3K36A double mutant in their analyses. By directly measuring the role of H3K36me, they may identify non-histone Set2 substrates important for UV-induced DNA damage repair. In addition, these analyses might indicate which level(s) of H3K36me are important for UV damage repair and/or recruitment of the repair machinery. The study would also be significantly strengthened by more rigorous analyses of yeast harboring H3K36A point mutations. CPD-seq for H3K36A, *rad16* H3K36A and *rad26* H3K36A mutant."

RESPONSE: In the revised manuscript, we have used CPD-seq to directly characterize the effects of the histone H3K36A point mutant on UV damage repair, as suggested by the Reviewer. The data indicate that the H3K36A mutant causes a defect in TC-NER relative to the matched H3 WT control, similar to that observed in the *set2*Δ mutant (i.e., compare Fig. 2A-C and Fig. 6C-E). Moreover, the H3K36A point mutant results in partial restoration of repair in a GG-NER defective *rad16*Δ mutant background of the non-transcribed strand (NTS) of yeast genes (i.e., Fig. 6F-H), to a similar extent as a *set2*Δ mutant. These findings directly implicate Set2-catalyzed H3K36 methylation in promoting canonical TC-NER and suppressing cryptic TC-NER of the NTS of yeast genes. We chose not to analyze H3K36me by ChIP-seq in response to UV damage, since the near-random distribution of UV damage across the genome would render it difficult to characterize coherent methylation patterns associated with UV damage and

repair in a heterogeneous population of UV-irradiated cells. The new H3K36A CPD-seq data are included in new Figure 6 and Supplemental Figures S9-S11 and described on pages 14-15 of the revised manuscript.

1. “In Fig. 1, the authors conclude that Set2-mediated H3K36me is required for resistance to UV damage. If this is the case, then one would expect a phenocopy when comparing UV sensitivity of *set2*Δ to H3K36A mutants. As presented, the data are not convincing that the point mutation of H3K36 demonstrates equal UV sensitivity to *set2*D mutants. Perhaps this would be more convincing if the data were quantified (as in Fig. 5). This experiment would also be strengthened by inclusion of a *set2*Δ H3K36A double mutant, as discussed above. Furthermore, as the data are presented, the reader cannot make a direct comparison of the *set2*Δ and H3K36A mutant strains since the experiments were performed on separate plates. At a minimum, the authors should repeat the experiment on the same plate to allow direct comparison of *set2*Δ and H3K36A mutants. Finally, the H3K36A mutant was not assayed for the kinetics of DNA repair (panels F and G) and should be included to bolster their conclusions.”

RESPONSE: We thank the Reviewer for these helpful suggestions. In order to directly compare the UV sensitivity of the *set2*Δ and H3K36A mutants in the same strain background, we created *set2*Δ in the WY499 background strain used for H3K36A. Comparing the UV sensitivity of the *set2Δ* mutant to H3K36A (new Supplemental Fig. S8) indicates that both mutants have similar UV sensitivity. Moreover, the *set2*Δ H3K36A double mutant did not show any additional UV sensitivity than either single mutant, indicating the two mutants are epistatic. These findings were confirmed by a quantitative UV survival assay (Supplemental Fig. S8B). We also quantified the kinetics of repair in the point mutant H3K36A using the T4 endonuclease V - alkaline gel analysis (new Fig. 6A,B), which revealed that H3K36A mutant had an similar overall effect on repair as the *set2*Δ mutant, with a marginal decrease in global repair activity at the 3hr post-UV time point. Taken together, these new findings support our model that Set2 methylation of H3K36 regulates both canonical TC-NER of the TS and suppresses cryptic TC-NER of the NTS. We discuss these new data on pages 14-15 of the revised manuscript.

2. In Fig. 1F and 1G, the authors quantify alkaline gels to measure global CPD repair conclude that there is a minimal difference in Set2-dependent repair at the 3-h time point. Since TC-NER occurs preferentially over GG-NER, these data seem inconsistent with their model that Set2 is important for TC-NER and antagonizes GG-NER. Some discussion of this result seems warranted.

RESPONSE: Our model is that Set2 promotes TC-NER of the transcribed strand (TS), but suppresses cryptic TC-NER of the non-transcribed strand (NTS) by preventing antisense transcription. Deletion of *SET2* may also have some effect on GG-NER, as hinted at in the 3hr repair time point by alkaline gel analysis and our CPD-seq data. We now discuss the finding that Set2 may also facilitate GG-NER on page 7, paragraph 2 and page 17, paragraph 1 of the revised manuscript.

3. “In Fig. 2D and E, the authors hypothesize that differences in Set2-dependent TC-NER should be reflected by plotting CPD-seq data alongside the transcription frequency of yeast genes. The analyses as presented utilizes the transcript levels of wild type yeast for analyses of *set2*Δ mutants. Since Set2 is a known regulator of transcription, the authors should repeat the *set2*Δ CPD repair analyses using transcript levels from *set2*Δ yeast mutants using available data from references 30-34.”

RESPONSE: The transcription frequency values are derived from a previously published analysis of an *rpb1-1* mutant strain [1], which provided genome-wide estimates of mRNA stability and transcription frequency that have been widely used in other studies. While similar data on transcription frequency are not available for the *set2*Δ mutant strain, we instead analyzed CPD-seq repair relative to changes in mRNA levels in a *set2*Δ mutant strain, using data from references 32-34, as suggested by the Reviewer. This analysis (see Fig. 2F) revealed that there was a general repair defect (particularly along the transcribed strand) in the *set2*Δ mutant strain, largely independent of the effects of *set2*Δ on mRNA levels. This new analysis is described on page 9, paragraph 2 and page 17, paragraph 2 of the revised manuscript.

4. “Fig. 2E shows that the majority of NTS CPDs still remain in the *set2Δ* mutant after 2 hours of repair. This result influences their $\log_2$ ratio analyses of the wild type and *set2*Δ mutant strains. It also suggests that Set2 impacts GG-NER when the wild type repair enzymes are present. Can the authors comment and/or clarify this result in the text? (Is this also related to the discrepancy in Set2 repair as described in Fig. 1G at t=3?) It could support a model in which the delayed TC-NER in Set2 mutants results in a delay to repair the NTS.”

RESPONSE: We agree that our data are consistent with the hypothesis that Set2 also impacts GG-NER, as indicated by higher fraction of CPDs remaining in the NTS in the *set2Δ* mutant. Because there can be variability in the absolute levels of unrepaired CPDs measured by CPD-seq due to differences in library prep, etc. (see page 7, paragraph 1), our WT and *set2*Δ CPD-seq data were normalized using the CPD levels measured by alkaline gel analysis (i.e., Fig. 1F-G). Hence, the Reviewer is correct that this likely contributes to this observation. We should note that a GG-NER defect in the *set2*Δ mutant strain could actually reduce the magnitude of its TC-NER defect, since slower GG-NER of the NTS would amplify asymmetry in repair of the TS relative to the NTS (e.g., see [2]). We have modified the text on pages 7-8, to discuss these issues and this interesting finding, as suggested by the Reviewer.

5. “In Fig. 3 the authors discuss the increase in unrepaired CPDs across the TS in *set2*Δ mutants. However, it seems that Set2 is important for CPD repair in general as there are more unrepaired CPDs across all genomic regions measured. For example, the wild type strain starts at a fraction of CPD remaining=0.5, while *set2Δ* = 0.6. Is the difference they are measuring relative to the starting number or is it more important to consider overall CPD repair? If these curves were presented on the same plot, this conclusion would might be clearer.”

RESPONSE: As mentioned above, our data suggest that the fraction of CPDs remaining in *set2Δ* mutant strain is somewhat higher than the WT, possibly due to slower overall repair. Hence, more unrepaired CPDs on the TS of yeast genes could reflect both the effects of slower TC-NER as well as GG-NER. To specifically examine the effects of Set2 on TC-NER, we analyzed to what extent there is more rapid repair on the TS relative to the NTS by calculating the $\log_2$ ratio of CPDs remaining on the TS relative to the NTS. This statistic essentially quantifies the amount of repair asymmetry between the TS and NTS due to TC-NER, which we have previously used to characterize TC-NER defects in mutants of the RSC ATP-dependent chromatin remodeling complex, which affected both GG-NER and TC-NER[3]. This analysis indicates that both the *set2*Δ (Fig. 2C and Sup. Fig. 2E,F) and H3K36A mutants (new Fig. 6E) cause a specific decrease in repair of the TS of yeast genes by the TC-NER pathway. Consistent with this hypothesis, the *set2*Δ *rad16*Δ double mutant also shows a defect in repair of the TS (Fig. 4D,E and Sup. Fig. S4), even though GG-NER is absent in this strain background. For these reasons, our data indicate that Set2 regulates TC-NER.

MINOR POINTS
1. “The title of the manuscript only considers the role of Set2 on TC-NER, yet there are also compelling results regarding the role of Set2 on GG-NER. The authors may wish to consider a title that more accurately depicts their complete dataset.”

RESPONSE: We have decided to retain the original title, since the focus of our study is on the role of Set2 in regulating both canonical TC-NER of the TS of yeast genes and cryptic TC-NER of the NTS. While we agree that our data suggest that Set2 may also regulate GG-NER, we hope to further investigate this phenomenon in a potential future study, since we believe additional data is likely necessary to rigorously establish the role and mechanism of Set2 in regulating the GG-NER pathway.

2. “A brief description of CPD-seq in the Results section would assist the reader in interpreting their results.”

RESPONSE: We thank the Reviewer for this suggestion, and have added a brief description of CPD-seq method on pages 6-7 of the revised manuscript.

3. “Can the authors clarify why they decided to use CPD-seq at the t=2 h time point? Their previous manuscript describing CPD-seq (Mao et al. 2016) indicates that strand-specific differences in CPD repair can be observed after only 20 minutes. It would be helpful for the reader to include some rationale for their decision on this time point.”

RESPONSE: The CPD-seq method measures repair by mapping unrepaired CPDs at the various repair time points and determining the fraction of CPDs that have been repaired/removed relative to the 0hr control. Although CPD removal can be seen in the TS at 20 minutes, especially for highly transcribed genes, the CPD-seq method more accurately measures repair at later time points (e.g., 2 or 3 hours) since more CPD removal has occurred over these longer time scales. For this reason, we have typically

used a 2hr time point to characterize TC-NER defects in *rad26*Δ [2] and *elf1*Δ [4]. We have included text describing the rationale for choosing these time points on page 21, paragraph 2 of the revised manuscript.

4. "Are Supplemental Fig. S2G and S2H showing the same data as Fig. 2D and E? It is unclear what the differences are between these datasets (it seems that the Fig S2G and S2H might be from the 3 h. time point)."

RESPONSE: Essentially, the data shown in Supplemental Figure S2G and S2H is an independent replicate experiment of the *set2*Δ mutant and WT CPD-seq data following 2hr (or 3hr) repair. Importantly, the data in Sup. Fig. S2 show very similar patterns to that of Figure 2, highlighting the reproducibility of our CPD-seq repair experiments. This is now clarified in the revised legend for Supplemental Fig. S2.

5. "Figs. 4D, 4E, 5A, and 5B line colors are not indicated. I'm assuming that red is TS and blue is NTS as described earlier in the manuscript, but this should be clarified."

RESPONSE: Yes, that is correct. We have now included this information in the respective Figure legends.

6. "Fig. 5C needs clarification related to what exactly the authors are measuring. More clearly labeling the figure could provide this clarification."

RESPONSE: Fig. 5C is measuring the difference in unrepaired CPDs between the *set2*Δ *rad16*Δ double mutant and the *rad16*Δ single mutant along the NTS and TS for yeast genes across the genome. We observe fewer unrepaired CPDs along the NTS of the *rad16*Δ *set2*Δ mutant, particularly for SRAT genes with antisense transcription (top of Fig. 5C). These data are now quantified in new Supplemental Fig. S5A and are more clearly described in the Fig. 5C legend.

7. "Pg. 10- Citation is missing for the requirement of Rad26 and Rad16 for TC- and GG-NER respectively."

REPONSE: The citations have been inserted.

8. "Pg. 11- The alkaline gel is indicated as Fig. 1D, but is actually Fig. 1F; Quantification of the data is indicated as Fig. 1E, but is actually Fig. 1G."

RESPONSE: We thank the reviewer for catching this error. We have corrected the figure numbers in the revised manuscript.

9. 'Pg. 15- The authors state that "there were fewer unrepaired CPDs along the NTS in the *rad16Δ set2Δ* double mutant relative to the *rad16* single mutant, indicating that *set2Δ* promotes repair of the NTS in the *rad16* background." This is confusing as it is written, since it is considering the impact of *set2* deletion mutations on Rad16-dependent GGNER. Perhaps it should be reworded to indicate the impact of Set2 on

CPD repair (ie Set2 inhibits repair of the NTS). Similar issues are found throughout the manuscript regarding conclusions about the absence of a gene rather than the function of the gene.'

RESPONSE: We have revised the text here and elsewhere in the manuscript to clarify our findings.

Reviewer 2:

"In this manuscript the authors examine the role of the yeast Set2 enzyme that methylates histone H3 lysine 36 in transcription coupled nucleotide-excision repair (TC-NER) and global genomic-nucleotide excision repair (GG-NER). They demonstrate that Set2 has a role in global TC-NER since a set2 mutant is UV sensitive, it is epistatic to the TC-NER factor rad26 mutant in the repair of UV-induced DNA damage, and set2 mutants have higher levels of unrepaired DNA damage genome-wide compared to wild-type cells. They also provide evidence that loss of Set2-mediated H3K36 methylation enhances antisense transcription and partially suppresses UV sensitivity of a mutant defective in GG-NER because it enhances repair of the non-coding transcribed strand (NTS) over the transcribed strand (TS). They provide evidence that one mechanism by which Set2 loss partially suppresses GG-NER repair defects is due to impaired recruitment of the RPD3S deacetylase complex whose epigenetic reader subunit Eaf3 binds Set2-mediated H3K36 methylation. They also provide genetic evidence that the ability of a set2 mutation to suppress GG-NER depends on the Dot1 histone methyltransferase, which is required for antisense transcription in set2 mutants. Overall, I found the manuscript to be well written and the work presented to be well-done and of potential interest to the readership of PLOS Genetics. However, I believe that while the data presented are of high-quality and provide new insight into the role that Set2-mediated H3K36 methylation plays in DNA repair through the TC-NER and GG-NER pathways, there are some important scientific deficiencies that still need to be addressed. Those scientific concerns are outlined below."

Scientific concerns

1. "The authors argue that Set2 loss causes global defects in TC-NER but suppresses GG-NER defects due to the increase in antisense transcription normally repressed by Set2. Are the DNA repair defects in TC-NER solely due to transcriptional interference that causes impaired Pol II transcription? Or does the physical presence of the antisense transcripts prevent repair? Could the authors combine their set2, rad26, rad16, set2/rad26, and set2/rad16 mutants with overexpression of the nuclear exosome subunit Rrp6 and/or the Xrn1 exonuclease to determine if this enhances TC-NER and/or GG-NER repair. Additionally, what happens to UV sensitivity and DNA repair of set2 and/or rad26 mutants are combined with rrp6 and/or xrn1 mutants?"

RESPONSE: To test the potential contribution of antisense transcripts themselves to UV sensitivity and repair, we have created mutants in *XRN1* or *RRP6* in wild type and

rad16Δ strain with or without *set2*Δ and tested for the UV sensitivity (Supplemental Fig. S7A-D). The results suggest that deletion of either *XRN1* or *RRP6* does not affect the UV sensitivity caused by *set2*Δ in wild type cells or the UV resistance imparted by *set2*Δ in a *rad16*Δ strain background. These results indicated that the physical presence of Set2-repressed antisense transcripts in these exonuclease mutants does not modulate UV sensitivity. Since loss of exonuclease activity did not modulate *set2*Δ UV sensitivity, we did not pursue additional overexpression or repair experiments. Interestingly, our data also indicate that the *xrn1*Δ deletion in a WT background caused a slight increase in UV resistance (Supplemental Fig. S7A). We hypothesize that this may be caused by Xrn1's role in modulating transcription initiation and elongation of its target genes or preventing backtracking of RNAPII [5-7]. In summary, these new results suggest that the physical presence of the antisense transcript likely does not contribute to the UV sensitivity phenotypes in the *set2*Δ mutant strains. We discuss these new findings on page 13 of the revised manuscript.

2. "The authors provide data that indicates loss of Set2-repressed antisense transcription promotes partial suppression of the GG-NER defect in the rad16 mutant. They indicate that impaired recruitment specifically of RPD3S (through the Eaf3 subunit) functions in this process as it increases antisense transcription. However, Set2 mediated H3K36 methylation also recruits additional chromatin effectors to transcribed genes, including the ATP-dependent chromatin remodeling Isw1b complex via the Ioc4 subunit. This complex has an important role in nucleosome remodeling that could be important for repair through these pathways. The authors have not addressed whether such additional chromatin effectors that depend on Set2 activity also are contributing to the TC-NER and/or GG-NER pathways. They should provide at least some genetic evidence that loss of Set2 dependent recruitment of these additional chromatin effectors is not contributing to the TC-NER and/or GG-NER repair process."

RESPONSE: To test the potential role of Isw1b/Ioc4 in Set2-regulated NER, we deleted *IOC4* in different *set2*Δ mutant backgrounds and measured their UV sensitivity. These data indicate that the *ioc4*Δ mutant does not modulate UV sensitivity in a WT or *rad16*Δ mutant background, either in the presence or absence of *SET2* (Supplemental Fig. S7E,F). These findings indicate that Set2-mediated recruitment of Isw1b complex likely does not play a role in TC-NER. We describe these new data on page 14, paragraph 1 of the revised manuscript.

Reviewer 3:

"In this study, the authors provide evidence for a role of Set2p/ H3 K36 methylation in promoting transcription-coupled nucleotide excision repair, NER, in response to UV damage in *S. cerevisiae*. In doing so, the authors provide genetic evidence demonstrates *SET2* functions in an *RAD26*-dependent pathway for transcription-coupled NER. Quite interestingly, they also demonstrate loss of *SET2* or H3 K36

mutation can partially suppress global genomic nucleotide excision repair defects in cells lacking *RAD16*. The authors link this phenotype to the activation of cryptic transcription of the non-coding strand due to loss of *SET2*/ H3 K36 methylation. Together, their results support a model in which this non-coding strand-derived gene expression promotes transcription coupled-NER (albeit, using a mechanism not requiring H3 K36 methylation for the repair itself.) Overall, the study's observations are thought provoking, but the study needs a fair amount of clarification."

Comments:

1. "In the section 'Set2 is important for TC-NER', the authors incorrectly refer to Fig. 1D and 1E when discussing experiments using T4 endonuclease V and alkaline gel electrophoresis to measure global repair of CPD lesions. It is unclear if that data is in Fig. 1F and 1G or simply missing. In the same section, it is unclear if Fig 1F and 1G data are part of a separate experiment, associated with CPDseq from Fig 2 or S2. Clarification is needed on how data shown in 1 figure relates to data shown in other figures throughout manuscript. Also, for several experiments, e.g. Figure 2 or S2, the authors comment they are reusing/reanalyzing/showing some replicates of WT and rad16Δ data from earlier published work. Authors should consult with editors to determine if any permissions are needed from previous journal prior to publications."

RESPONSE: We thank the Reviewer for catching this error. The data are indeed shown in Fig. 1F,G. These experiments measure overall repair of CPD lesions using the T4 endoV - alkaline gel assay, and are completely independent of the CPD-seq experiments shown in Fig. 2, etc. However, we do use the overall repair values derived from the alkaline gel assays to normalize some of the CPD-seq data sets, as described in the methods. We have tried to clarify this more in the revised manuscript text. Also, the Reviewer is correct that the CPD-seq data for one of the wild-type replicates and the *rad16Δ* control have been previously published by us. Since we do not reuse any specific figures (but instead just re-analyzed the data), we do not believe any specific permissions are required, since re-analysis of genomics data is a standard practice in the field, just so long as the original publication is cited.

2. "For several figures related to the CPDseq analyses, beginning with Fig. 2A & B the authors 'bin' the data. Clarification for the reader is needed on how 'binning' was done. Also, for different CPDseq analyses, the authors state `~5000' or "~5200" genes were analyzed. By what criteria was this subset of genes chosen for analysis? The logic for the subset is unclear."

RESPONSE: In essence, the transcribed region of each gene (i.e., from the transcription start site (TSS) to transcription end site (TES)) was divided up into 6 bins, and repair was analyzed in each bin. Due to the variable lengths of the transcribed region for different genes, this analysis allowed us to present a summary of the average repair of many genes across the whole transcribed region. We also analyzed repair in 3 bins upstream and downstream of each gene. These bins had a fixed width of 167 bp.

For the bin analysis, we analyzed repair for ~5000 genes that had a well-defined TSS and TES (which we determined from coordinates for the 3' polyadenylation site) using data from reference [8]. We also analyzed repair at single-nucleotide resolution around the TSS. In this case, there were TSS coordinates for ~5200 yeast genes. The reason for the differences in the number of genes is that ~200 genes with TSS coordinates did not have corresponding TES coordinates in the referenced study [8], so they could not be included in the bin analysis. Note, we used bin analysis for analyzing repair along the length of entire genes, since differences in gene lengths made it impractical to analyze repair at single nucleotide resolution. We have expanded our discussion to clarify these points in the figure legends and in the revised methods (page 21-22).

3. **"**For the experiments in which Fraction of CPDs remaining (Fig. 3, Fig. S3), are mapped with respect to published nucleosome positions in WT cells, what evidence do the authors have, or is there in the literature, that nucleosome positioning has been conserved in *set2*, *rad16* or *rad16* set2 mutants relative to WT? While the authors may ultimately be correct, as presented, the alternate interpretation of this data is, e.g. there is a genome-wide "randomization" of nucleosome positioning in the rad16 mutants (e.g. do CPDs, when not removed, destabilize nucleosomes?). Or, for WT cells, when there is a correlation of elevated CPDs remaining at nucleosome positions, versus linker regions, could this mean linker regions are preferentially repaired in WT, but this preference is lost in the absence of global genomic NER? If the observed pattern is indeed transcription-dependent, will it be lost in a pol II quickstop mutant or upon treatment with actinomycin? More context for understanding the significance of the patterns would be helpful for the reader."

RESPONSE: We have previously shown using our CPD-seq that repair is slower in nucleosomal DNA and faster in adjacent linker regions in WT cells [9, 10], but this pattern is abolished in *rad16*Δ mutant cells [9]. It is theoretically possible that the loss of this nucleosome pattern in repair could be in part due to a 'genome-wide randomization of nucleosome positions in the rad16 mutants', as suggested by the Reviewer. However, a previous study has shown that deletion of *RAD16* causes little if any change in nucleosome positioning in yeast both in unirradiated and UV-irradiated cells [11]. Hence, the loss of nucleosome-associated pattern of repair in the *rad16*Δ cells simply reflects the loss of GG-NER activity, which produces this pattern of repair in nucleosomes in the first place. Similarly, a recent report indicates that the *set2*Δ mutant does not cause a significant change in nucleosome positioning relative the TSS of yeast genes [12]. For these reasons, we feel it is appropriate to analyze the repair data relative to WT nucleosome map, with the caveat there may be minor alterations in nucleosome positioning/occupancy in each mutant. These issues are now briefly discussed on page 22 of the revised manuscript.

4. "Please also clarify as to whether cells were grown/incubated in the dark for all experiments in this study after exposing cells to UV (e.g. photoreactivation promotes preferential rapid repair in linker DNA vs. nucleosomal DNA.)"

RESPONSE: Cells are incubated in dark for repair, CPD-seq, and UV sensitivity assays, as indicated in the methods section (see pages 20-21). Hence, photoreactivation should not contribute to the observed repair patterns.

5. "Fig 4D, 5A and B, please label the transcribed vs non-transcribed strands in the figure key. Is the Data in S3A and B, the same data as is shown in Fig 4 D, but just plotted onto the same graph? If so, S3A and S3B should be removed from the manuscript. Regardless, how the datasets in the figures relate to each other should be clarified."

RESPONSE: As mentioned above (see response to Reviewer 1), we now include in the Figure 4 and 5 legends text indicating that blue indicate NTS, and red indicate TS, as in the other figures. The Reviewer is correct that Fig. S3A and B is the same as that shown in Fig. 4D, and we have removed these figure panels at the recommendation of the Reviewer.

6. "In Fig 5, the authors compare evaluate CPDs at a subset of Set2-repressed antisense transcripts STRATs. By what criteria were these 463 STRATs chosen for analysis? PolyA? Is this also seen for other STRATs? The logic for choosing to only look at this subset of STRATs (and whether a specific kind of cryptic NTS transcript is important) is unclear."

RESPONSE: We originally chose the 463 polyA SRATs since these are antisense transcripts that are induced in yeast cells with a deletion in *SET2* [13]. We have also performed similar analysis using the complete list of SRATs (see Sup. Fig. S6 and S11), which yielded similar results. These new analyses are discussed on pages 12 and 15 of the revised manuscript.

7. "Reference for cryptic transcription in *set2* cells requiring *DOT1* in last paragraph prior to Discussion actually refers to a mammalian DOT1L study. Please check all references for accuracy."

RESPONSE: We thank the Reviewer for pointing out this error. We have inserted the correct reference and double-checked all other references for accuracy.

8. "Perhaps the authors could comment on the prevalence of cryptic transcripts at genes in the *set2* mutants and how that relates to dose of damage, and subsequent repair. Clearly, loss of *SET2* confers resistance to UV to cells lacking *RAD16* on the level of 10-100x, depending on dose, in growth assays and to a lesser degree by 3 hr in their T4 EndoV assays. But, do the authors have a sense of the level of CBDs being produced/KB or /ORF at the doses being provided, and the level or frequency of cryptic transcription that is occurring to drive repair in the absence of global genomic NER and photolyase-driven repair? Would this need to be happening at a large scale for viability, and not just at genes having overlapping PolyA STRATs? Does the 'back of the envelope' calculation make sense? Or, is cryptic transcription itself what is important in the absence of *RAD16*, not necessarily that this transcription is *set2*-derived? If true,

then other factors that normally suppress cryptic transcription should also suppress the UV sensitivity of *rad16* mutants when deleted."

RESPONSE: This is a great question, because our data suggest that although repair of the NTS is somewhat faster in polyA or all SRAT-containing genes, it also occurs at a slower frequency throughout the genome in the *set2*Δ mutant, including at non-SRAT genes. Hence, we suspect that low-levels of antisense transcription are present in non-SRAT genes in the *set2*Δ mutant. Moreover, UV irradiation may exacerbate antisense transcription in the *set2*Δ mutant, perhaps by transiently disturbing chromatin structure during NER. We agree that it will be important to test whether other factors that suppress cryptic transcription (e.g., Spt16, Spt6, etc.) also promote UV resistance in the absence of *RAD16*, which we hope to do in future studies. We also hope to pursue future studies looking at whether UV irradiation promotes cryptic transcription. We discuss these issues on pages 12 and 19 of the revised manuscript.

Reviewer 4

"This study examines the role of Set2 histone methyltransferase in nucleotide excision repair using the elegant genome-wide CPD mapping method that they developed. They demonstrate that a Set2 mutation reduces transcription and leads to elevated antisense transcription with the overall consequence of reduced transcription-coupled repair in the transcribed strand and increased repair in the nominally non-transcribed strand. They also show that this aids in the survival of mutants defective in global repair (Rad16). While this is a plausible scenario, the data is not very compelling, and I have three specific concerns."

1. "First, the authors have not shown increased UV resistance or increased global repair of CPDs in Set2Rad16 double mutant verses the Rad16 single mutant (Fig.1)."

RESPONSE: The Reviewer is correct that this data is not shown in Figure 1; instead, it is shown in Figures 4 and 5. Figure 4A shows that UV resistance of *RAD16* deletion strain is significantly increased in the *rad16*Δ *set2*Δ double mutant relative to the *rad16*Δ mutant alone. Similar results are shown in Figure 5E, in which deletion of *SET2* or *EAF3* increase UV resistance of the *rad16*Δ mutant. These results were confirmed by a quantitative UV sensitivity assay (Fig. 5G), which confirmed that deletion of either *SET2* or *EAF3* increases the UV resistance of the *rad16*Δ mutant. Moreover, eliminating the Set2-catalyzed methylation site in H3K36 (i.e., H3K36A) also increases UV resistance in the *rad16*Δ mutant (Figure 4B). Finally, we show that this UV resistance requires the Rad26 TC-NER factor, as there is no increase in UV resistance when *RAD26* is also deleted (Figures 4C and 5F). Taken together, these results demonstrate that deletion of *SET2* or mutation of its methylation site in histone H3 increases the UV resistance of a *rad16*Δ mutant strain. We clarify that this UV resistance data is in Fig. 4A (not Fig. 1) in the revised text on page 6, paragraph 1.

Second, our CPD-seq data indicate that there is faster repair of the NTS of the *rad16*Δ *set2*Δ double mutant relative to *rad16*Δ alone (Figures 4D and 5A-C). This repair

is quantified in Supplemental Fig. S5. Moreover, we now include new data showing that the H3K36A mutant, which eliminates Set2-catalyzed H3K36 methylation, also promotes repair of the NTS in a *rad16*Δ mutant background (new Figure 6F,H and Supplemental Figs. S10 and S11). Note, that we do not argue that *set2*Δ promotes global repair in a *rad16*Δ mutant, but instead promotes cryptic TC-NER of the NTS of yeast genes, particularly those with Set2-repressed antisense transcripts (SRATs).

2. "Second, the various genomic plots showing transcribed verses non-transcribed strands only show marginal differences, and such differences might be expected for a strain containing a mutation in a gene known to be involved in regulating several cell cycle and division genes (Cell Reports 20:2693)."

RESPONSE: Our data indicate that the *set2*Δ mutant causes UV sensitivity in yeast that is epistatic with a deletion in the TC-NER factor *RAD26*, indicating that it functions in TC-NER. Our CPD-seq data of multiple independent *set2*Δ replicates confirms that repair of the transcribed strand (TS) of yeast genes is reduced relative to the non-transcribed strand, consistent with a defect in TC-NER. We acknowledge that TC-NER is not abolished in the *set2*Δ mutant, but instead is decreased. This is consistent with the fact that Set2 is not a core NER (or TC-NER) factor, but instead likely promotes TC-NER through a chromatin-based mechanism. Moreover, our new CPD-seq data indicates that the H3K36A mutant, which eliminates Set2-catalyzed methylation, also causes a similar TC-NER defect (Figure 6). These results support our model that Set2 regulates TC-NER in yeast.

It is important to note that the *Cell Reports* paper indicating that Set2 regulates cell cycle and DNA replication genes (referenced by the Reviewer above) was a study of the function of Set2 in *S. pombe*, not *S. cerevisiae*. Since *S. pombe* and *S. cerevisiae* have significant differences in cell cycle regulation, chromatin, transcription regulation and repair (among other differences), it is not clear how applicable these results are to our study. To address the possible effects of gene expression changes in the *set2*Δ mutant on repair, we now include a new analysis, in which we analyzed the set2Δ-dependent changes in repair along the TS (and NTS) for genes sorted by their change in mRNA levels in the *set2*Δ mutant (Figure 2F). This new analysis indicates that deletion of *SET2* effects repair across the genome, largely independent of any gene expression change. This new analysis is described on page 9, paragraph 2 and page 17, paragraph 2 of the revised manuscript.

3. "Finally, there is no convincing quantitative data measuring the levels of the presumed anti-sense transcription. Thus, I recommend that this manuscript is better suited for a journal with a more specialized readership."

RESPONSE: It has been well established through multiple studies (e.g., [13-16]) that Set2 methylation regulates cryptic transcription in yeast genes, including antisense transcription. For our analysis, we used lists of genes that have been identified as containing cryptic antisense transcripts that are induced upon deletion of *SET2* (i.e., SRAT genes). These SRAT genes were based on quantitative analysis of RNA-seq data from *set2*Δ mutant cells in yeast [13]. Since the levels of Set2-repressed antisense

transcription are already well established in the literature, we believe it is outside the scope of our paper to re-address this question. Instead, we simply use these gene lists to confirm that repair of the NTS in the *set2*Δ *rad16*Δ double mutant is especially prominent in genes containing a Set2-repressed antisense transcript, consistent with our model.

Finally, we would like to thank each of the Reviewers for their helpful comments and suggestions. Their efforts have helped to significantly improve the manuscript.

Sincerely,

John J. Wyrick

(on behalf of the authors)

References

1. Holstege FC, Jennings EG, Wyrick JJ, Lee TI, Hengartner CJ, Green MR, et al. Dissecting the regulatory circuitry of a eukaryotic genome. Cell. 1998; 95: 717-28. PMID: 9845373.

2. Duan M, Selvam K, Wyrick JJ, Mao P. Genome-wide role of Rad26 in promoting transcription-coupled nucleotide excision repair in yeast chromatin. Proceedings of the National Academy of Sciences of the United States of America. 2020; 117: 18608-16. PMID: 32690696.

3. Bohm KA, Hodges AJ, Czaja W, Selvam K, Smerdon MJ, Mao P, et al. Distinct roles for RSC and SWI/SNF chromatin remodelers in genomic excision repair. Genome research. 2021; 31: 1047-59. PMID: 34001524.

4. Geijer ME, Zhou D, Selvam K, Steurer B, Mukherjee C, Evers B, et al. Elongation factor ELOF1 drives transcription-coupled repair and prevents genome instability. Nature Cell Biology. 2021; 23: 608-19.

5. García-Martínez J, Pérez-Martínez ME, Pérez-Ortín JE, Alepuz P. Recruitment of Xrn1 to stress-induced genes allows efficient transcription by controlling RNA polymerase II backtracking. RNA biology. 2021; 18: 1458-74. PMID: 33258404.

6. Begley V, Jordán-Pla A, Peñate X, Garrido-Godino AI, Challal D, Cuevas-Bermúdez A, et al. Xrn1 influence on gene transcription results from the combination of general effects on elongating RNA pol II and gene-specific chromatin configuration. RNA biology. 2021; 18: 1310-23. PMID: 33138675.

7. Begley V, Corzo D, Jordán-Pla A, Cuevas-Bermúdez A, Miguel-Jiménez L, Pérez-Aguado D, et al. The mRNA degradation factor Xrn1 regulates transcription

elongation in parallel to Ccr4. Nucleic acids research. 2019; 47: 9524-41. PMID: 31392315.

8. Park D, Morris AR, Battenhouse A, Iyer VR. Simultaneous mapping of transcript ends at single-nucleotide resolution and identification of widespread promoter-associated non-coding RNA governed by TATA elements. Nucleic acids research. 2014; 42: 3736-49. PMID: 24413663.

9. Mao P, Smerdon MJ, Roberts SA, Wyrick JJ. Asymmetric repair of UV damage in nucleosomes imposes a DNA strand polarity on somatic mutations in skin cancer. Genome research. 2020; 30: 12-21. PMID: 31871068.

10. Mao P, Smerdon MJ, Roberts SA, Wyrick JJ. Chromosomal landscape of UV damage formation and repair at single-nucleotide resolution. Proceedings of the National Academy of Sciences of the United States of America. 2016; 113: 9057-62. PMID: 27457959.

11. van Eijk P, Nandi SP, Yu S, Bennett M, Leadbitter M, Teng Y, et al. Nucleosome remodeling at origins of global genome-nucleotide excision repair occurs at the boundaries of higher-order chromatin structure. Genome research. 2019; 29: 74-84. PMID: 30552104.

12. Eriksson PR, Clark DJ. The yeast ISW1b ATP-dependent chromatin remodeler is critical for nucleosome spacing and dinucleosome resolution. Scientific reports. 2021; 11: 4195. PMID: 33602956.

13. Venkatesh S, Li H, Gogol MM, Workman JL. Selective suppression of antisense transcription by Set2-mediated H3K36 methylation. Nature communications. 2016; 7: 13610. PMID: 27892455.

14. Carrozza MJ, Li B, Florens L, Suganuma T, Swanson SK, Lee KK, et al. Histone H3 methylation by Set2 directs deacetylation of coding regions by Rpd3S to suppress spurious intragenic transcription. Cell. 2005; 123: 581-92. PMID: 16286007.

15. Churchman LS, Weissman JS. Nascent transcript sequencing visualizes transcription at nucleotide resolution. Nature. 2011; 469: 368-73. PMID: 21248844.

16. Lee S, Oh S, Jeong K, Jo H, Choi Y, Seo HD, et al. Dot1 regulates nucleosome dynamics by its inherent histone chaperone activity in yeast. Nature communications. 2018; 9: 240. PMID: 29339748.
