# Supplementary material for: Set2 histone methyltransferase regulates transcription coupled-nucleotide excision repair in yeast
Source: PLoS Genet. 2022 Mar 9;18(3):e1010085. doi: 10.1371/journal.pgen.1010085 (PMC8936446; doi:10.1371/journal.pgen.1010085)
Supplement: Attachment — Submitted filename: PGenetics D 21 01151 review.docx [file pgen.1010085.s016.docx]

Selvam K PLOS GENETICS

In this study, the authors provide evidence for a role of Set2p/ H3 K36 methylation in promoting transcription-coupled nucleotide excision repair, NER, in response to UV damage in *S. cerevisiae*. In doing so, the authors provide genetic evidence demonstrates *SET2* functions in an *RAD26*-dependent pathway for transcription-coupled NER. Quite interestingly, they also demonstrate loss of *SET2* or H3 K36 mutation can partially suppress global genomic nucleotide excision repair defects in cells lacking *RAD16*. The authors link this phenotype to the activation of cryptic transcription of the non-coding strand due to loss of *SET2*/ H3 K36 methylation. Together, their results support a model in which this non-coding strand-derived gene expression promotes transcription coupled-NER (albeit, using a mechanism not requiring H3 K36 methylation for the repair itself.) Overall, the study’s observations are thought provoking, but the study needs a fair amount of clarification.

Comments:

1. In the section “Set2 is important for TC-NER”, the authors incorrectly refer to Fig. 1D and 1E when discussing experiments using T4 endonuclease V and alkaline gel electrophoresis to measure global repair of CPD lesions. It is unclear if that data is in Fig. 1F and 1G or simply missing. In the same section, it is unclear if Fig 1F and 1G data are part of a separate experiment, associated with CPDseq from Fig 2 or S2. Clarification is needed on how data shown in 1 figure relates to data shown in other figures throughout manuscript. Also, for several experiments, e.g. Figure 2 or S2, the authors comment they are reusing/reanalyzing/showing some replicates of WT and *rad16*Δ data from earlier published work. Authors should consult with editors to determine if any permissions are needed from previous journal prior to publications.
2. For several figures related to the CPDseq analyses, beginning with Fig. 2A & B the authors ‘bin’ the data. Clarification for the reader is needed on how ‘binning’ was done. Also, for different CPDseq analyses, the authors state `~5000’ or “~5200” genes were analyzed. By what criteria was this subset of genes chosen for analysis? The logic for the subset is unclear.
3. For the experiments in which Fraction of CPDs remaining (Fig. 3, Fig. S3), are mapped with respect to published nucleosome positions in WT cells, what evidence do the authors have, or is there in the literature, that nucleosome positioning has been conserved in *set2*, *rad16* or *rad16* set2 mutants relative to WT? While the authors may ultimately be correct, as presented, the alternate interpretation of this data is, e.g. there is a genome-wide “randomization” of nucleosome positioning in the rad16 mutants (e.g. do CPDs, when not removed, destabilize nucleosomes?). Or, for WT cells, when there is a correlation of elevated CPDs remaining at nucleosome positions, versus linker regions, could this mean linker regions are preferentially repaired in WT, but this preference is lost in the absence of global genomic NER? If the observed pattern is indeed transcription-dependent, will it be lost in a pol II quickstop mutant or upon treatment with actinomycin? More context for understanding the significance of the patterns would be helpful for the reader.
4. Please also clarify as to whether cells were grown/incubated in the dark for all experiments in this study after exposing cells to UV (e.g. photoreactivation promotes preferential rapid repair in linker DNA vs. nucleosomal DNA.)
5. Fig 4D, 5A and B, please label the transcribed vs non-transcribed strands in the figure key. Is the Data in S3A and B, the same data as is shown in Fig 4 D, but just plotted onto the same graph? If so, S3A and S3B should be removed from the manuscript. Regardless, how the datasets in the figures relate to each other should be clarified.
6. In Fig 5, the authors compare evaluate CPDs at a subset of Set2-repressed antisense transcripts STRATs. By what criteria were these 463 STRATs chosen for analysis? PolyA? Is this also seen for other STRATs? The logic for choosing to only look at this subset of STRATs (and whether a specific kind of cryptic NTS transcript is important) is unclear.
7. Reference for cryptic transcription in *set2* cells requiring *DOT1* in last paragraph prior to Discussion actually refers to a mammalian DOT1L study. Please check all references for accuracy.
8. Perhaps the authors could comment on the prevalence of cryptic transcripts at genes in the *set2* mutants and how that relates to dose of damage, and subsequent repair. Clearly, loss of *SET2* confers resistance to UV to cells lacking *RAD16* on the level of 10-100x, depending on dose, in growth assays and to a lesser degree by 3 hr in their T4 EndoV assays. But, do the authors have a sense of the level of CBDs being produced/KB or /ORF at the doses being provided, and the level or frequency of cryptic transcription that is occurring to drive repair in the absence of global genomic NER and photolyase-driven repair? Would this need to be happening at a large scale for viability, and not just at genes having overlapping PolyA STRATs? Does the ‘back of the envelope’ calculation make sense? Or, is cryptic transcription itself what is important in the absence of *RAD16*, not necessarily that this transcription is *set2*-derived? If true, then other factors that normally suppress cryptic transcription should also suppress the UV sensitivity of *rad16* mutants when deleted.
